# Supplementary material for: Mixed Weyl semimetals and low-dissipation magnetization control in insulators by spin–orbit torques
Source: Nat Commun. 2017 Nov 14;8:1479. doi: 10.1038/s41467-017-01138-7 (PMC5684220; doi:10.1038/s41467-017-01138-7)
Supplement: Supplementary file 1 — Supplementary Information [file 41467_2017_1138_MOESM1_ESM.pdf]

SUPPLEMENTARY FIGURES

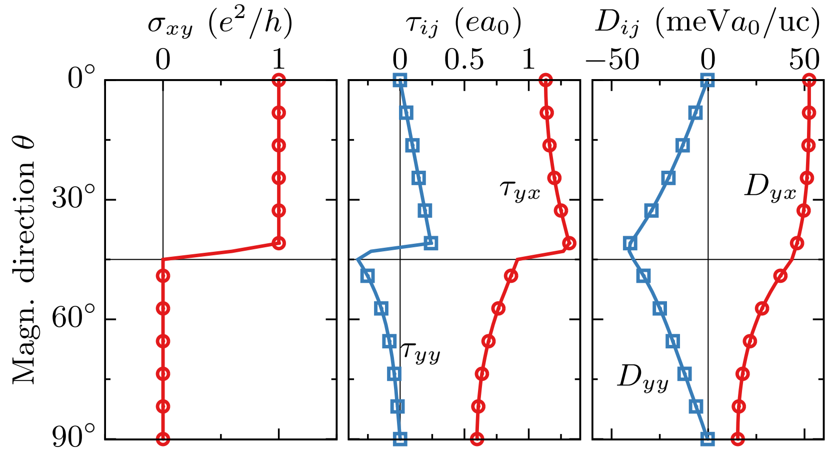

Supplementary Figure 1. **Dependence on magnetization direction in semi-hydrogenated Bi bilayer.** The anomalous Hall conductivity σ_{xy}, the torkance τ_{ij}, and the spiralization D_{ij} at the actual Fermi level as a function of the magnetization direction $\hat{\mathbf{m}} = (\sin\theta, 0, \cos\theta)$. A mixed Weyl point emerges in the electronic structure at $\theta = 43°$.

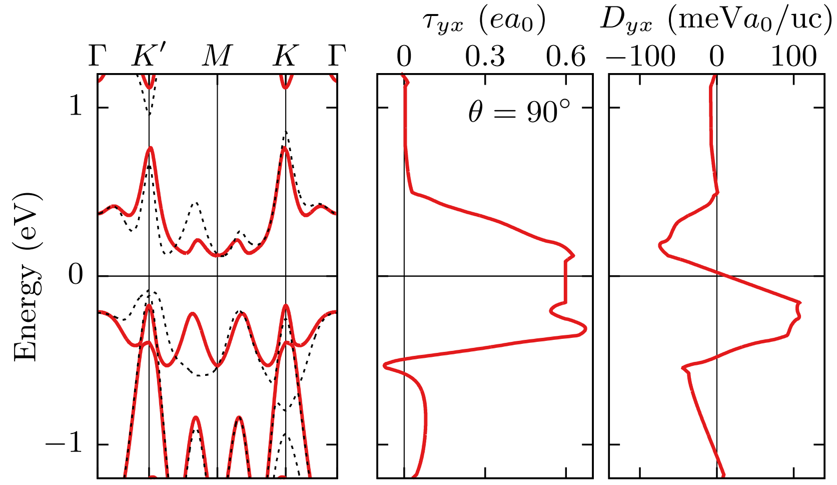

Supplementary Figure 2. **Semi-hydrogenated Bi bilayer with in-plane magnetization.** Band structure, torkance τ_{yx}, and spiralization D_{yx} as function of the position of the Fermi level if the magnetization is parallel to the film, i.e., $\theta = 90°$. For comparison, dashed lines in the left panel denote the spectrum for an out-of-plane magnetization ($\theta = 0°$).

SUPPLEMENTARY NOTES

Supplementary Note 1 — GaBi film with exchange field

Using the FLEUR code (see www.flapw.de), we performed self-consistent electronic structure calculations of an intrinsically non-magnetic GaBi film, employing the generalized gradient approximation and a plane-wave cut-off of $4.0\,a_0^{-1}$, where a_0 is Bohr's radius. The in-plane lattice constant was $8.5\,a_0$ and we chose a muffin tin radius of $2.45\,a_0$ for both atom species. Subsequently, we constructed 16 maximally-localized Wannier functions out of 32 energy bands with the frozen window extending up to $2\,\mathrm{eV}$ above the Fermi level. Finally, in order to substantiate the predicted effect of monopole-driven spin-orbit torques, the exchange term $\frac{1}{2}\mathbf{B}\cdot\boldsymbol{\sigma}$ was added to the corresponding tight-binding Hamiltonian, where $\boldsymbol{\sigma}$ is the vector of Pauli matrices and $\mathbf{B} = B_0(\sin\theta, 0, \cos\theta)$ denotes the imposed exchange field.

Supplementary Note 2 — Anisotropy with magnetization direction in semi-hydrogenated Bi bilayer

In Supplementary Fig. 1, we show the dependence of anomalous Hall conductivity, torkance, and spiralization on the magnetization direction $\hat{\mathbf{m}} = (\sin\theta, 0, \cos\theta)$ in the semi-hydrogenated bismuth film. For general magnetization directions, both torkance and spiralization display also small non-zero components τ_{yy} and D_{yy}, respectively, since the shape of these response tensors is dictated by the crystal symmetries and not due to Onsager's reciprocity relations. When the Weyl point emerges in the electronic structure at $\theta = 43°$, the system undergoes a topological phase transition from a Chern insulator to a trivial magnetic insulator, which is accompanied by a jump in σ_{xy} and a similar drop of τ_{ij}. As apparent from Supplementary Figs. 1 and 2, the torkance τ_{yx} is still remarkably prominent in the regime of the trivial insulator, i.e., for $\theta > 43°$.
